# Book Review: Speech Perception, Production and Acquisition: Multidisciplinary Approaches in Chinese Languages

**DOI:** 10.3389/fpsyg.2021.621481

**Published:** 2021-05-10

**Authors:** Jiaqiang Zhu, Xiaoxiang Chen, Fei Chen

**Affiliations:** School of Foreign Languages, Hunan University, Changsha, China

**Keywords:** Chinese lexical tones, acoustic information, phonological information, music-language relationship, multimodel processing

In recent decades, a surge of interest in Chinese languages has been observed among scholars worldwide, yet a question remains to be rarely asked: why do we research Chinese languages? *Speech Perception, Production and Acquisition: Multidisciplinary approaches in Chinese languages* gives us an implicational account concerning the role played by Chinese languages, which belong to tone languages including Mandarin (see Figure 1) and other major dialects (e.g., Cantonese and Southern Min).

**Figure 1:**
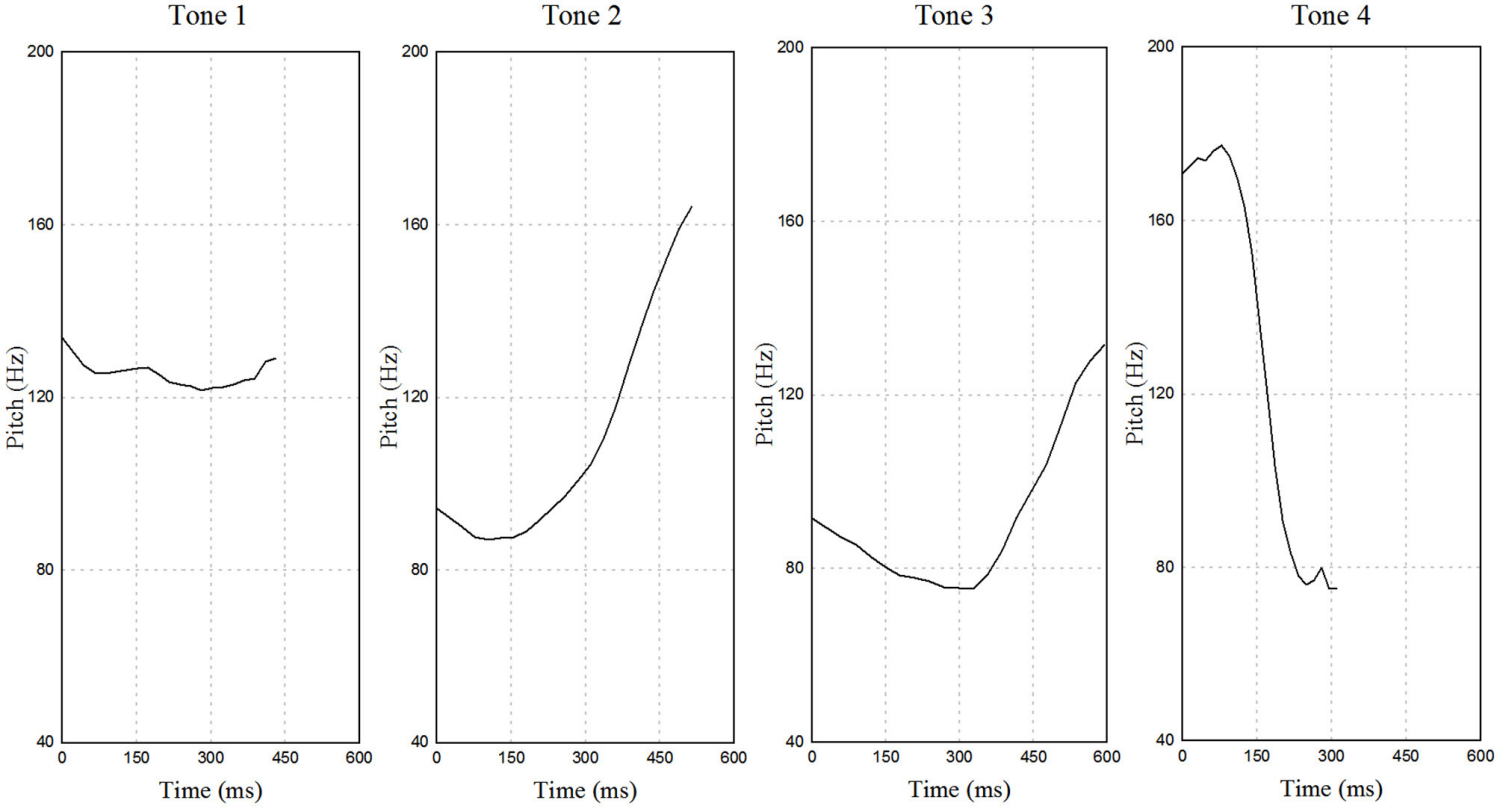
Time by frequency representations of four Mandarin lexical tones borne on monosyllable /pa/ uttered by a male native speaker.

Edited by Huei-Mei Liu, Feng-Ming Tsao, and Ping Li, this book is the latest complete overview of the novel and interdisciplinary field of studies for Chinese, with particular reference to research on lexical tones which has offered intensive insights in areas of linguistics, psychology, education, cognitive neuroscience, and communication disorders. The book brings together four main parts incorporating 12 chapters, each of which provides specific research topics and methodologies written by the leading figures. The first part addresses general acoustic analyses of lexical tones mainly using behavioral approaches. With the development of non-invasive neuroimaging techniques, the second part reveals neural representations of lexical tones consistent with the results of behavioral methods to support models of speech processing. The next part of this book pinpoints multimodal perception of lexical tones via visual and auditory cues, with the finding of domain-general transfer across music and language. The final part extends the research scope to developmental trajectories of learning Chinese from infancy throughout childhood, combining with an illustration of a clinical group who suffers from autism spectrum disorder.

Summarizing the entire book could be difficult, but it is worthwhile to highlight a few authors' perspectives to probe into some entrenched issues from innovative angles. First, there is a collection of wonderful studies on acoustic and phonological processing of lexical tones relating to hemispheric lateralization (Chapter 3 by Chao-Yang Lee and Seth Wiener, and Chapter 5 by Keke Yu, Ruiming Wang, and Ping Li). Divergent from prior studies examining lexical tone perception as a whole, the recently growing studies have shown that two types of information of lexical tones, namely acoustic information and phonological information, are processed differentially by our brain as evidenced by their respective neurophysiological correlates (Yu et al., 2019). Second, a heated debate has been discussed as regards the relationship between music and language (Chapter 8 by Jia Hoong Ong, Shen Hui Tan, Alice H. D. Chan, and Francis C. K. Wong). Separating acoustic and phonological information helps corroborate that music associates with speech, as bidirectional transfers can be estimated in a full spectrum (i.e., lower-level pitch perception and higher-level categorical processing). Lexical tones offer the logic and optimal testing ground for such an issue, because pitch is verified as a primary acoustic cue for lexical tone processing; meanwhile, according to *Music, Language, and the Brain* (Patel, 2008), pitch also serves as the basis for music. This shared cue with its overlapping neural substrates across domains iterates the possibility for driving reciprocal influences between music and language such that a musical advantage in lexical tone processing is frequently reported (Zhu et al., 2021). Third, one more interesting topic of Chinese refers to multimodal perception of lexical tones (Chapter 9 by Yue Wang, Joan A. Sereno, and Allard Jongman). Dating back to the 1950s, many studies devoted to connecting visual cues with auditory listening, especially for segmental processing. Compared with most segmental phonemes, lexical tones are produced by intricate articulatory vocal cord gestures, which involve glottal and sub-glottal activities independent of vocal tract configurations (Howie, 1976). In this regard, it may not be easy to conceive that visual facial cues can be linguistically meaningful and enhance lexical tone perception. However, despite the “invisible” property of lexical tones, there appears to be empirical evidence showing that movements of the head, neck, jaw, eyebrows, and lips are linked to lexical tone processing such that the degree of benefits of visual cues corresponds to the extent of pitch contour movement dynamicity and shape contrastivity of specific lexical tones (Connell et al., 2013).

This book triggers several thoughts in our minds. First, we draw a more explicit picture than before in the light of lexical tones. Although typological differences of a tone language vs. a non-tonal language are well-documented, lexical tones have received increasing attention because of the special position as the phonemically contrastive suprasegmental feature (Yip, 2002). It is not surprising that second language listeners of Chinese struggle to integrally process lexical tones and segments, and map this combination into a previously stored mental lexicon (Wiener and Lee, 2020). Second, it necessitates that brain science be applied to experiments for the reason that neural responses faithfully reflect behavioral performance. As uncovered by an event-related potential study, English learners of Mandarin exhibited greater attentional resource allocation in the perception of lexical tones, albeit they showed native-like behavioral performance (Shen and Froud, 2019). This discipline not only encourages researchers to explore neural underpinnings of lexical tone processing but aids therapists to properly treat clinical populations with disabilities.

Essentially, as an effective update of researching Chinese languages, a key factor of this book's strength and usefulness lies in its extensive reviewing of literature and opinion in an interdisciplinary manner. This book differs from traditional studies of first language acquisition or language teaching and learning, because it focuses on learning, perception, and production of speech from native preverbal infants to non-native adult learners of Chinese. The research of speech processing in the Chinese context is a large and rapidly growing field, thus readers, regardless of their backgrounds and intentions, can considerably benefit from this most recent review of this book. After going through the entire book, readers are deemed to have a better understanding of Chinese as the most widely spoken tone language in the world (Gu et al., 2012). In future studies, it is important to promote diversity (linguistics, ethnics, and geography) and intervention in translational research (converting research to training and practice) in terms of speech processing and learning of Chinese among both typical and atypical populations.

## Author Contributions

JZ and FC chose the book together. JZ drafted the manuscript. XC and FC revised the manuscript. All authors have approved the final version of the manuscript.

## Conflict of Interest

The authors declare that the research was conducted in the absence of any commercial or financial relationships that could be construed as a potential conflict of interest.
